# Prognostic value of platelet to lymphocyte ratio (PLR) in breast cancer patients receiving neoadjuvant therapy: a systematic review and meta-analysis

**DOI:** 10.3389/fimmu.2025.1658571

**Published:** 2025-08-20

**Authors:** Ziqian Zhao, Haoyi Xu, Binlin Ma, Chao Dong

**Affiliations:** The Clinical Medical Research Center of Breast and Thyroid Tumor in Xinjiang, Tumor Hospital Affiliated to Xinjiang Medical University, Urumqi, China

**Keywords:** platelet to lymphocyte ratio (PLR), breast cancer, NACT, prognostic value of survival, meta-analysis

## Abstract

**Background:**

The platelet to lymphocyte ratio(PLR) is widely recognized as an important biomarker of systemic inflammation and has been associated with treatment responses in breast cancer (BC) patients undergoing neoadjuvant therapy. However, existing evidence remains inconsistent. This meta-analysis aims to systematically investigate the prognostic value of PLR in BC patients receiving neoadjuvant chemotherapy (NACT).

**Methods:**

A broad and systematic search of the literature was carried out using PubMed, Embase, Web of Science, and the Cochrane Library, covering all available records from the inception of each database through April 7, 2025. Study selection was guided by a set of predetermined inclusion and exclusion parameters. Primary outcomes included overall survival (OS), disease-free survival (DFS), and pathological complete response (pCR), assessed through hazard ratios (HRs) or odds ratios (ORs) with corresponding 95% confidence intervals (CIs).

**Results:**

Twenty-four studies involving 7,557 BC patients receiving NACT were included. Elevated PLR was significantly associated with reduced pCR rates (HR = 1.51; 95% CI: 1.24–1.84; p < 0.0001; I² = 70%), shorter OS (HR = 1.64; 95% CI: 1.27–2.11; p = 0.0002; I² = 0%), and decreased DFS (HR = 2.29; 95% CI: 1.54–3.39; p < 0.0001; I² = 44%). Subgroup analyses indicated that PLR’s prognostic value varied by timing of PLR measurement, geographic location, and PLR cutoff values.

**Conclusions:**

Elevated PLR is significantly correlated with poorer clinical outcomes in BC patients undergoing NACT, suggesting its potential as a predictive biomarker for treatment efficacy. However, due to methodological limitations of the included studies, further prospective investigations are required to confirm these findings across diverse populations.

**Systematic review registration:**

https://www.crd.york.ac.uk/prospero/, identifier CRD420251064051.

## 1 Introduction

Breast cancer constitutes the globe’s second most frequently diagnosed malignancy, as reported in the 2022 Global Cancer Statistics compiled by the World Health Organization’s International Agency for Research on Cancer. Breast cancer is responsible for an estimated 2.297 million newly diagnosed cases each year, maintaining its status as the leading cancer type affecting women globally. In China alone, the yearly incidence is projected to be around 357,000 cases. This figure accounts for 15.6% of all newly diagnosed malignancies among women (1). With the continuous advancement of therapeutic approaches, such as surgical intervention, radiotherapy, chemotherapy, endocrine therapy, immunotherapy, and targeted therapy—the survival outcomes for patients with BC have improved substantially (2). At the point of initial diagnosis, an estimated 5% to 15% of individuals are found to have breast cancer that has progressed to a locally advanced stage. For this subgroup, the five-year survival rate remains low, estimated at only around 29% (3). NACT is currently recognized as a standard therapeutic approach for the management of locally advanced breast cancer. It is widely regarded as both an effective and evidence based treatment strategy (4). The main goals of NACT are multifaceted. These include reducing the overall tumor burden, downstaging axillary lymph node involvement, and increasing the feasibility of surgical resection. Additionally, NACT aims to enhance the chances of achieving successful breast-conserving surgery (5). Achieving a pCR is regarded as the most desirable outcome of neoadjuvant therapy, serving as a surrogate indicator of improved long-term prognosis. Nonetheless, current research has highlighted significant discrepancies in pCR rates among the various molecular subtypes of breast cancer. Notably, individuals diagnosed with HER2-positive breast cancer tend to achieve a pathological complete response in approximately 30% of cases following treatment with NACT. For patients diagnosed with triple-negative breast cancer, the documented rates of pathological complete response vary between 30% and 50%. Conversely, individuals with tumors that are positive for estrogen receptor (ER) expression but lack HER2 amplification tend to have markedly reduced pathological complete response rates, generally falling below 10% (6, 7). These observed differences in treatment response may be explained by distinct molecular alterations within the tumor microenvironment. Various elements, including the density of stromal tumor-infiltrating lymphocytes (TILs), the expression of cyclin-dependent kinases, and the activity of non-coding RNA transcripts, have been shown to influence how breast cancer patients respond to NACT (8). However, these predictive factors are often difficult to obtain in clinical settings. Thus, there is a pressing need for a cost-effective, practical, and easily accessible method to predict the response to NACT.

Systemic inflammatory responses are widely recognized as key contributors to the progression of BC (9). Research has shown that systemic inflammatory indicators, most notably the platelet to lymphocyte ratio(PLR), are linked to clinical outcomes and therapeutic efficacy in various types of cancer (9–11). As a readily obtainable and cost-effective blood-based marker, an elevated PLR has been associated with poorer prognostic outcomes in breast cancer and may act as an indicator of diminished responsiveness to NACT (12). In a single-center study by Li et al, 215 breast cancer patients who received NACT followed by surgery were enrolled. After a ten-year follow-up, a higher pre-NACT PLR was found to be predictive of reduced OS (13). In another multicenter study, 63 breast cancer patients who underwent NACT between 2018 and 2024 were retrospectively analyzed by Fiste et al. A higher pre-NACT PLR was found to be predictive of a lower pCR (14). Despite emerging evidence, the utility of PLR as a predictive biomarker for tailoring neoadjuvant therapeutic approaches in breast cancer remains insufficiently defined and warrants further comprehensive investigation. While earlier meta-analyses have validated the prognostic relevance of PLR in forecasting OS, DFS, and pCR among breast cancer patients receiving NACT, further investigation is still warranted (12), they included only studies published before 2022. Subsequently, a considerable volume of additional clinical research has emerged in the literature, yet their findings remain inconclusive. Accordingly, this meta-analysis incorporates an additional nine studies published between 2022 and 2025 (13–21). In light of the ongoing discrepancies among recent findings, the present analysis seeks to extend prior research by incorporating up-to-date evidence to more comprehensively assess the prognostic significance of PLR in breast cancer patients undergoing NACT.

## 2 Materials and methods

### 2.1 Literature search

This meta-analysis was performed in alignment with the methodological framework detailed in the 2020 update of the Preferred Reporting Items for Systematic Reviews and Meta-Analyses (PRISMA) guidelines (22). Moreover, the research protocol was submitted in advance and formally recorded in the International Prospective Register of Systematic Reviews (PROSPERO), bearing the registration ID CRD420251064051.

Two researchers (ZQZ and CD) independently formulated the search methodology used in this study. Both investigators identified and selected relevant subject headings and keywords to conduct a comprehensive literature search across PubMed, Embase, Web of Science, and the Cochrane Library, covering publications from database inception to April 7, 2025. The search terms included “Blood Platelet,” “Platelets,” “Platelet,” “Thrombocytes,” “Thrombocyte,” “Lymphocyte,” “Lymphoid Cells,” “Lymphoid Cell,” “Breast Neoplasm,” “Breast Tumor,” “Breast Cancer,” “Breast Malignant Neoplasm,” “Mammary Cancers,” “Neoadjuvant Therapies,” “Neoadjuvant Chemotherapy Treatments,” “Neoadjuvant Systemic Therapy,” “Neoadjuvant Radiation,” and “Neoadjuvant Radiation Treatments.” A comprehensive description of the search methodology can be found in S1.

### 2.2 Study selection

Eligibility for study inclusion was determined according to the following predefined criteria (1): pathological confirmation of breast cancer (2); receipt of neoadjuvant therapy (3); evaluation of the prognostic value of PLR concerning OS, DFS, or pCR (4); availability or calculability of HRs, ORs, and corresponding 95% CIs (5); classification into elevated and reduced PLR cohorts based on explicitly stated threshold values; and (6) full-text availability.

Exclusion criteria included (1): secondary literature and non-primary sources, including review articles, editorial commentaries, conference summaries, individual case studies, and correspondence pieces (2); studies that did not provide adequate information to calculate HRs or corresponding 95% CIs (3); studies not reporting relevant survival outcomes; and (4) duplicate or overlapping publications.

Titles and abstracts were independently reviewed by two authors (ZQZ and CD), who also evaluated the full texts of potentially eligible for inclusion in the analysis. Any discrepancies were addressed and resolved through consensus-based discussion.

### 2.3 Data extraction

Two reviewers (ZQZ and CD) independently carried out the process of data extraction. Any conflicts in interpretation were addressed collaboratively through collective discussion, with all contributing authors participating to reach a unified agreement. The collected data encompassed various study characteristics, including first author’s name, publication year, study location, design, sample size, patient demographics, duration of study, treatment approach, TNM stage, PLR cutoff values, timing of PLR measurement, and reported HRs or ORs with 95% CIs for OS, DFS, and pCR.

### 2.4 Quality assessment

Study quality was assessed using the Newcastle–Ottawa Scale (NOS), which validates methodological rigor across three key domains: cohort selection, cohort comparability, and the determination of outcomes. The highest possible rating that can be assigned using the Newcastle–Ottawa Scale is 9 points (23).

### 2.5 Statistical analysis

Pooled HRs or ORs with corresponding 95% CIs were calculated to assess the prognostic significance of PLR in breast cancer patients undergoing NACT. Statistical heterogeneity was evaluated using Cochran’s Q test and Higgins’ I² statistic (24), with significant heterogeneity defined as I² >50% or P < 0.1. All analyses utilized a random-effects model to account for between-study variability. To test the stability of the aggregated outcomes, sensitivity analyses were performed. Additionally, subgroup analyses were carried out to identify possible heterogeneity sources and verify findings related specifically to OS, DFS and pCR. A separate random-effects model was applied to each subgroup to obtain subgroup-specific hazard ratios and odds ratios. Publication bias was visually assessed through funnel plots and statistically evaluated using Egger’s regression test, considering a p <0.05 indicative of significant bias. All statistical procedures were executed using STATA version 15.0 and Review Manager (Rev Man) version 5.4. The strength of evidence for each outcome was appraised following the Grading of Recommendations, Assessment, Development, and Evaluation (GRADE) framework, with results categorized into one of four tiers: high, moderate, low, or very low (25).

## 3 Results

### 3.1 Study characteristics

**Figure 1:**
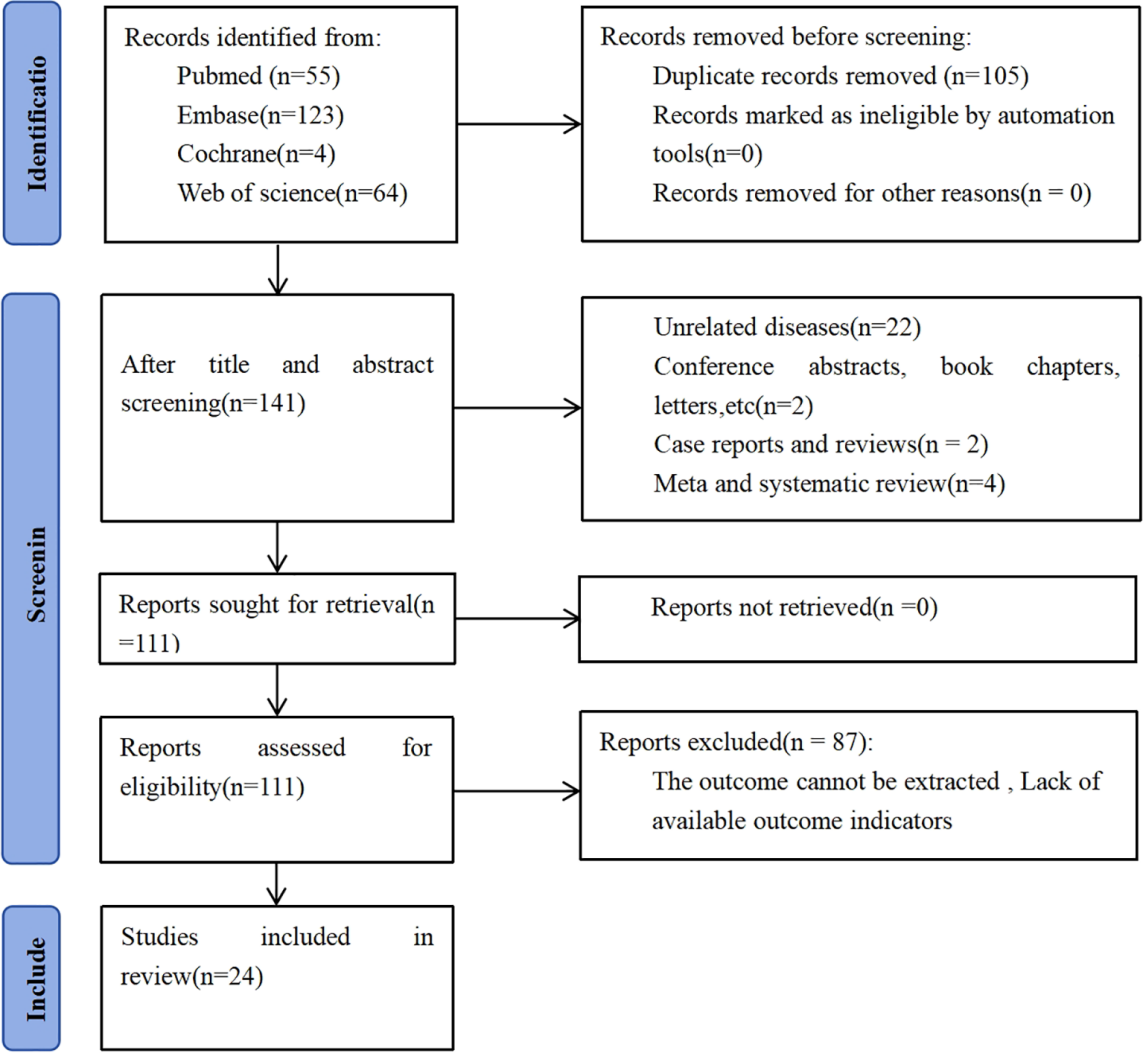
Flow chart of literature screening.

An initial total of 246 records were retrieved from database searches. Following the elimination of 105 duplicate entries, 141 distinct studies were retained for further evaluation. Screening titles and abstracts resulted in excluding 30 articles. Detailed assessment of the full texts from the remaining 111 studies led to the exclusion of 87 articles, primarily due to inadequate data for survival analysis. In the end, 24 studies satisfied the inclusion criteria and were incorporated into the meta-analysis, encompassing a combined cohort of 7,557 individuals diagnosed with breast cancer. Participant enrollment across individual studies ranged from a minimum of 55 to a maximum of 1,994 individuals, as illustrated in **Figure 1**.

From the 24 studies published between 2016 and 2025, a total of 26 distinct comparison cohorts were derived for analysis. Of these, 18 comparison groups were conducted in Asia (9, 13, 15, 18–21, 26–35), 5 in Europe (16, 17, 36–38), and the remaining 3 were multicenter studies (14, 39). Among them, 25 comparison groups were retrospective in design (9, 13–21, 26, 27, 29–39), while the remaining 1 was prospective (28). All identified comparison cohorts were reported in English-language publications, with study durations spanning from 1996 to 2024. Two comparison groups did not report the study period (16, 38). The median age across these comparison groups ranged from 36 to 56.6 years. All patients received NACT, including 11 groups that received NACT alone, 14 groups that received NACT followed by surgery, and 1 group that received NACT combined with anti-HER2 therapy. For analytical purposes, patients were stratified into two cohorts based on PLR levels: those with elevated PLR and those with lower PLR values. Regarding PLR measurement, 22 comparison groups assessed PLR before NACT, while 4 evaluated PLR after NACT. Based on PLR assessment, 8 groups investigated its prognostic impact on OS, 9 on DFS, and 13 on pCR. **Table 1** presents a comprehensive overview of the key characteristics associated with each included comparison group.

**Table 1:** Basic characteristics of the included literature.

Author	Study period	Region	Study design	Population	Treatment method	Timing of detection	No. of patients	Mean/median age	TNM stage	PLR cut-off	Outcomes
Li 2024 (13)	2011-2023	China	Retrospective cohort	NACT and surgery	NACT and surgery	Before neoadjuvant therapy	215	36	I-III	130.7	OS/PCR
Chen 2024 (18)	2012-2023	China	Retrospective cohort	HER2 positive	NACT and anti-HER2 therapy	Before neoadjuvant therapy	744	48	I-IV	NA	PCR
Graziano 2019 (36)	1999-2018	Italy	Retrospective cohort	Early or locally advanced BC	NACT	Before neoadjuvant therapy	373	50	I-IV	104.5	PCR
Dan 2023 (21)	2012-2017	China	Retrospective cohort	NACT	NACT	After neoadjuvant therapy	257	50	II-III	NA	PCR
Van Berckelaer 2021 (39)	1996-2016	Multicenter	Retrospective cohort	Inflammatory BC	NACT and surgery	After neoadjuvant therapy	125	56.6	III	171	OS/DFS
Van Berckelaer 2021 (39)	1996-2016	Multicenter	Retrospective cohort	Inflammatory BC	NACT and surgery	Before neoadjuvant therapy	125	56.6	III	163	OS/DFS
Song 2022 (26)	2016-2018	China	Retrospective cohort	NACT and surgery	NACT and surgery	Before neoadjuvant therapy	144	50.4 (46.2-54.6)	I-III	158.4	DFS
Fiste 2024 (14)	2018-2024	Multicenter	Retrospective cohort	NACT	NACT	Before neoadjuvant therapy	63	52.6	I-III	NA	PCR
Corbeau 2020 (27)	2005-2013	China	Retrospective cohort	NACT	NACT	Before neoadjuvant therapy	280	50.3	NA	150	OS
Alan 2020 (28)	2015-2019	Turkey	Prospective cohort	Locally advanced BC	NACT	Before neoadjuvant therapy	55	48.5	II-III	225.3	PCR
Şahin 2021 (9)	2008-2019	Turkey	Retrospective cohort	NACT	NACT	Before neoadjuvant therapy	743	48	I-III	131.8	PCR
Ma 2021 (29)	2017-2018	China	Retrospective cohort	NACT and surgery	NACT and surgery	Before neoadjuvant therapy	203	46.6 (37.2-56.0)	II-III	135	DFS
Al Jarroudi 2021 (37)	2010-2014	Africa	Retrospective cohort	Inflammatory BC	NACT	Before neoadjuvant therapy	102	49 (37-61)	NA	178	OS/DFS
Ma 2023 (19)	2019-2022	China	Retrospective cohort	NACT and surgery	NACT and surgery	Before neoadjuvant therapy	112	51.0 (42.5-59.4)	NA	161.5	PCR
Asano 2016 (30)	2007-2013	Japan	Retrospective cohort	NACT and surgery	NACT and surgery	Before neoadjuvant therapy	177	56	IIA/IIIA	150	DFS
Kusama 2023 (20)	2013-2019	Turkey	Retrospective cohort	TNBC	NACT and surgery	Before neoadjuvant therapy	266	52.5	NA	180	PCR
Wang 2024 (17)	2013-2022	American	Retrospective cohort	NACT and surgery	NACT and surgery	Before neoadjuvant therapy	1994	50	I-III	103.6	PCR
Acikgoz 2022 (31)	2014-2019	Turkey	Retrospective cohort	Locally advanced BC	NACT	After neoadjuvant therapy	139	45	II-III	181.7	PCR
Jiang 2022 (32)	2012-2016	China	Retrospective cohort	NACT and surgery	NACT and surgery	Before neoadjuvant therapy	280	49	II-III	155	OS
Jiang 2022 (32)	2012-2016	China	Retrospective cohort	NACT and surgery	NACT and surgery	After neoadjuvant therapy	280	49	II-III	148	OS
Jin 2022 (33)	2014-2019	China	Retrospective cohort	NACT	NACT	Before neoadjuvant therapy	67	50	NA	106.3	PCR
Truffi 2022 (38)	NA	Italy	Retrospective cohort	NACT and surgery	NACT and surgery	Before neoadjuvant therapy	217	52 (41-63)	NA	152.5	DFS
Faur 2025 (16)	NA	Romania	Retrospective cohort	NACT	NACT	Before neoadjuvant therapy	142	50	NA	120.5	PCR
Kim 2019 (34)	2009-2017	South Korea	Retrospective cohort	NACT	NACT	Before neoadjuvant therapy	105	51.1 (41.6-60.6)	NA	143.4	DFS
Jiang 2020 (35)	2014-2018	China	Retrospective cohort	NACT and surgery	NACT and surgery	Before neoadjuvant therapy	249	51	NA	88.2	OS
Zhu 2025 (15)	2015-2022	China	Retrospective cohort	TNBC	NACT and surgery	Before neoadjuvant therapy	100	52	NA	152.1	DFS

NA refers to no available data.

### 3.2 Study quality

All 26 comparison groups had NOS scores ranging from 6 to 9 (**Supplementary Table S2**).

### 3.3 Meta-analysis results

#### 3.3.1 PLR and OS

**Figure 2:**
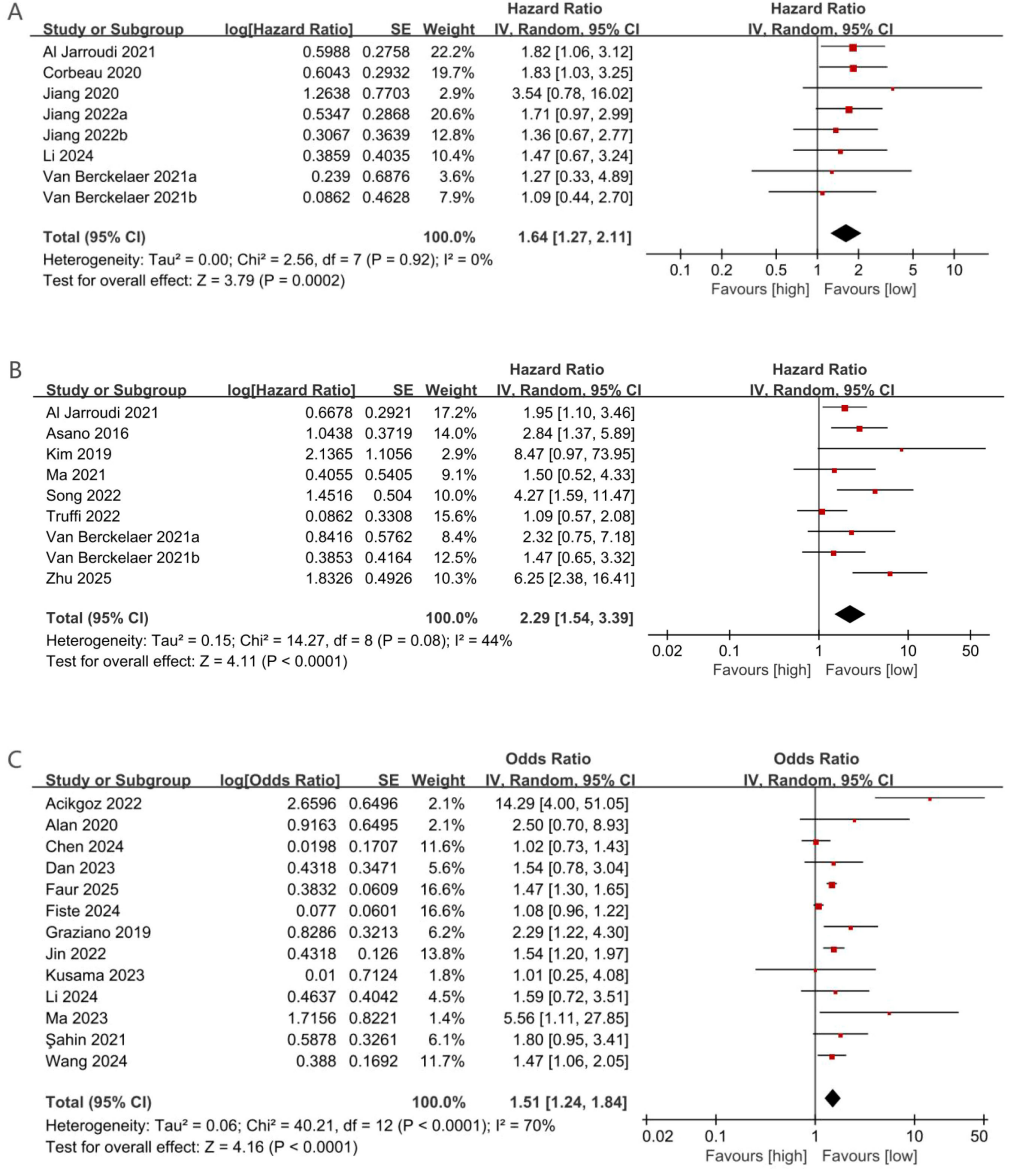
**(A)** Forest plots for the association between PLR and OS; **(B)** Forest plots for the association between PLR and DFS; **(C)** Forest plots for the association between PLR and pCR.

To examine the relationship between PLR and OS, eight comparison cohorts comprising a total of 1,656 patients were evaluated. Six studies evaluated PLR before NACT, while two studies measured PLR afterward. Overall, elevated PLR significantly correlated with reduced OS (HR = 1.64; 95% CI: 1.27–2.11; p = 0.0002). Subgroup analyses indicated significant prognostic value for pre-NACT PLR (HR = 1.70; 95% CI: 1.29–2.25; p = 0.0002; **Figure 2A**), whereas post-NACT PLR showed no significant association (HR = 1.34; 95% CI: 0.71–2.52; p = 0.36). Further stratification by median age demonstrated significant associations in both age groups: ≥50 years (HR = 1.65; 95% CI: 1.07–2.55; p = 0.02) and <50 years (HR = 1.63; 95% CI: 1.19–2.23; p = 0.002). Geographic subgroup analyses showed elevated PLR significantly predicted poorer OS in Asian (HR = 1.68; 95% CI: 1.23–2.30; p = 0.001) and European populations (HR = 1.82; 95% CI: 1.06–3.12; p = 0.03), but not in multicenter groups (HR = 1.14; 95% CI: 0.54–2.43; p = 0.73). Analysis by PLR cutoff values revealed significant prognostic value at thresholds ≥150 (HR = 1.67; 95% CI: 1.24–2.24; p = 0.0007), whereas lower thresholds did not yield significant results (HR = 1.56; 95% CI: 0.94–2.57; p = 0.08). Detailed results are summarized in **Table 2**.

**Table 2:** Pooled HRs/ORs for OS, DFS and pCR in subgroup analyses.

Subgroup	OS				DFS				PCR			
	Study group	HR [95%CI]	*P* value	*I* ^2^	Study group	HR [95%CI]	*P* value	*I* ^2^	Study group	OR [95%CI]	*P* value	*I* ^2^
Total	8	1.64 [1.27, 2.11]	0.0002	0%	9	2.29 [1.54, 3.39]	<0.0001	44%	13	1.51 [1.24, 1.84]	<0.0001	70%
Timing of detection												
Before neoadjuvant therapy	6	1.70 [1.29, 2.25]	0.0002	0%	8	2.31 [1.49, 3.56]	0.0002	51%	11	1.41 [1.19, 1.67]	<0.0001	62%
After neoadjuvant therapy	2	1.34 [0.71, 2.52]	0.36	0%	1	2.32 [0.75, 7.18]	0.14	NA	2	4.38 [0.50, 38.75]	0.18	89%
Mean/median age												
≥50y	4	1.65 [1.07, 2.55]	0.02	0%	7	2.58 [1.51, 4.41]	0.0005	56%	8	1.44 [1.18, 1.74]	0.0002	69%
<50y	4	1.63 [1.19, 2.23]	0.002	0%	2	1.84 [1.11, 3.04]	0.02	0%	5	2.14 [1.06, 4.32]	0.03	77%
Region												
Asia	5	1.68 [1.23, 2.30]	0.001	0%	5	3.45 [2.08, 5.73]	<0.00001	19%	9	1.77 [1.23, 2.55]	0.002	62%
Europe	1	1.82 [1.06, 3.12]	0.03	NA	2	1.49 [0.84, 2.63]	0.17	42%	3	1.49 [1.33, 1.66]	<0.00001	62%
multicenter	2	1.14 [0.54, 2.43]	0.73	0%	2	1.72 [0.89, 3.33]	0.11	0%	1	1.08 [0.96, 1.22]	0.2	NA
PLR cut-off												
≥150	5	1.67 [1.24, 2.24]	0.0007	0%	7	2.30 [1.49, 3.54]	0.0002	51%	4	3.78 [1.20, 11.87]	0.02	64%
<150	3	1.56 [0.94, 2.57]	0.08	0%	2	2.72 [0.54, 13.67]	0.22	49%	6	1.50 [1.36, 1.66]	<0.00001	0%

NA refers to no available data.

#### 3.3.2 PLR and DFS

PLR data pertaining to disease-free survival (DFS) were available in nine studies; eight of these evaluated PLR levels prior to NACT, while one study assessed PLR following NACT. An increased PLR demonstrated a strong inverse association with disease-free survival (DFS), with a pooled hazard ratio of 2.29 (95% CI: 1.54–3.39; p < 0.0001; **Figure 2B**). Subgroup analyses revealed significant associations only for pre-NACT PLR (HR = 2.31; 95% CI: 1.49–3.56; p = 0.0002), not post-NACT PLR (HR = 2.32; 95% CI: 0.75–7.18; p = 0.14). Significant prognostic value was found across median age groups (≥50 years: HR = 2.58; 95% CI: 1.51–4.41; p = 0.0005; <50 years: HR = 1.84; 95% CI: 1.11–3.04; p = 0.02). Subgroup analysis based on geographic region demonstrated a statistically significant association within Asian populations (HR = 3.45; 95% CI: 2.08–5.73; p < 0.00001), whereas no meaningful correlation was observed in European cohorts (HR = 1.49; 95% CI: 0.84–2.63; p = 0.17) or in studies conducted across multiple centers (HR = 1.72; 95% CI: 0.89–3.33; p = 0.11). PLR cutoff analysis showed significance at ≥150 (HR = 2.30; 95% CI: 1.49–3.54; p = 0.0002) but not at lower cutoffs (HR = 2.72; 95% CI: 0.54–13.67; p = 0.22). Results are presented in **Table 2**.

#### 3.3.3 PLR and pCR

Thirteen studies (5,170 patients) assessed PLR’s relationship with pCR, showing significantly lower pCR rates associated with elevated PLR (HR = 1.51; 95% CI: 1.24–1.84; p < 0.0001; **Figure 2C**). Pre-NACT PLR significantly correlated with reduced pCR (HR = 1.41; 95% CI: 1.19–1.67; p < 0.0001), while post-NACT PLR showed no significant effect (HR = 4.38; 95% CI: 0.50–38.75; p = 0.18). Subgroup analyses by median age confirmed significant associations across both age groups (≥50 years: HR = 1.44; 95% CI: 1.18–1.74; p = 0.0002; <50 years: HR = 2.14; 95% CI: 1.06–4.32; p = 0.03). PLR significantly predicted lower pCR rates in Asian (HR = 1.77; 95% CI: 1.23–2.55; p = 0.002) and European groups (HR = 1.49; 95% CI: 1.33–1.66; p < 0.00001), but not in multicenter groups (HR = 1.08; 95% CI: 0.96–1.22; p = 0.20). High PLR cutoff values (≥150) significantly correlated with lower pCR rates (HR = 3.78; 95% CI: 1.20–11.87; p = 0.02), whereas lower cutoffs did not yield significant results (HR = 1.50; 95% CI: 1.36–1.66; p < 0.00001). Results are detailed in **Table 2**.

### 3.4 Sensitivity analysis

**Figure 3:**
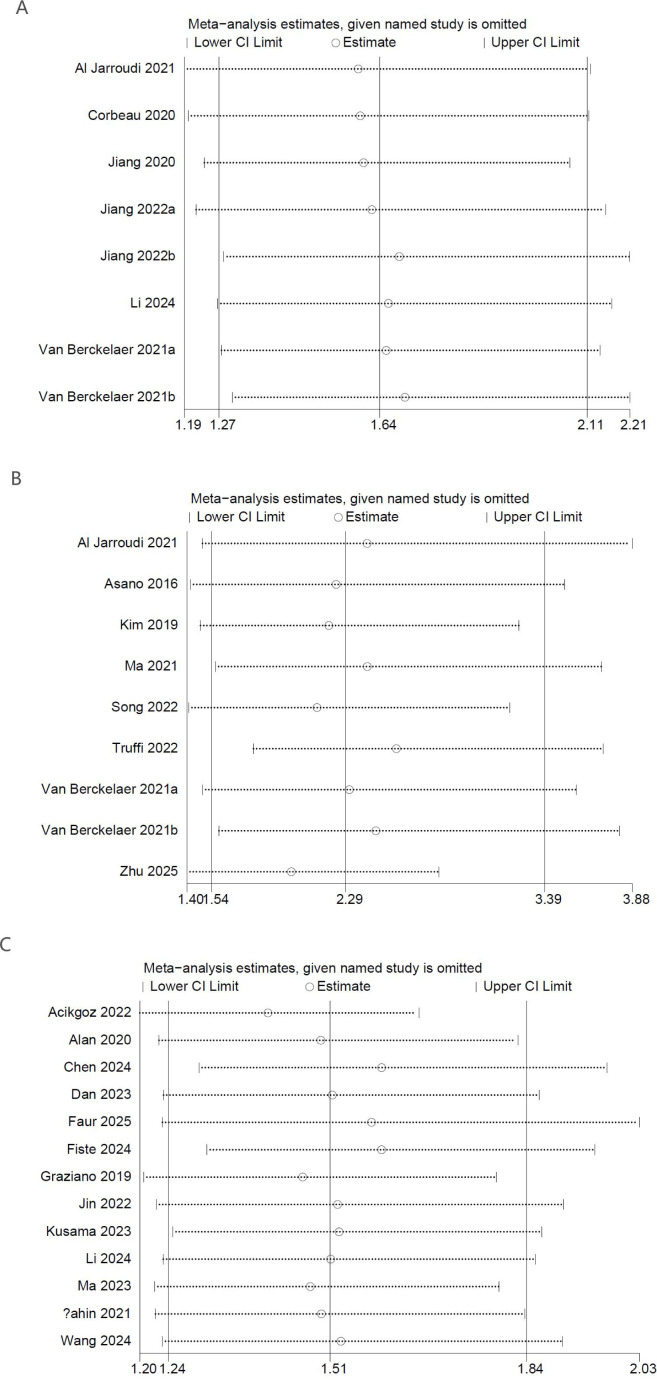
Sensitivity analysis of **(A)** OS, **(B)** DFS and **(C)** pCR.

To evaluate the reliability of the results related to PLR levels measured before NACT, a sensitivity analysis was performed. The stepwise removal of individual studies from the analysis had minimal impact on the overall pooled estimates, which consistently fell within the bounds of the original confidence intervals. The findings suggest that no individual comparison group exerted an undue influence on the results for OS (**Figure 3A**), DFS (**Figure 3B**), or pCR (**Figure 3C**), thereby supporting the consistency and robustness of the overall analysis.

### 3.5 Publication bias

**Figure 4:**
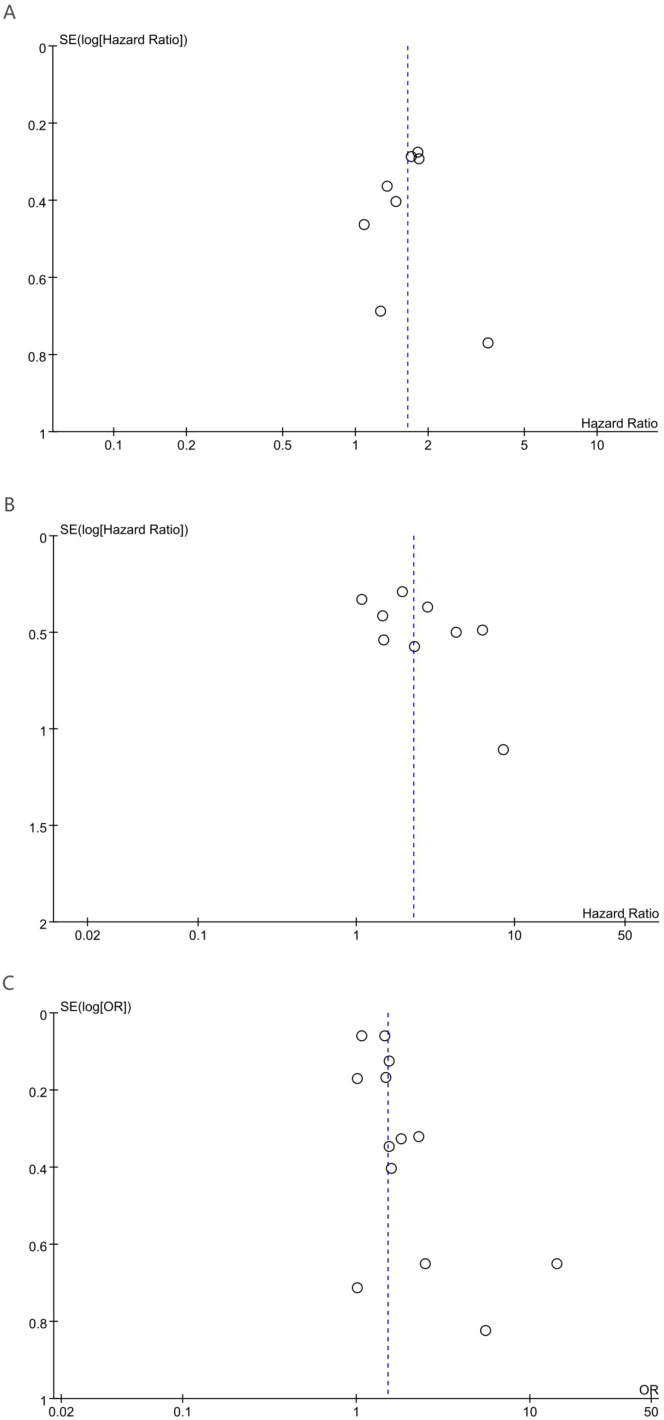
Funnel plot for the evaluation of publication bias for **(A)** OS, **(B)** DFS and **(C)** pCR.

Potential publication bias was evaluated through visual inspection of funnel plots alongside statistical assessment using Egger’s regression analysis. Egger’s regression analysis indicated no statistically significant signs of publication bias for OS (p = 0.85), DFS (p = 0.146), or pCR (p = 0.053). Additionally, funnel plot symmetry supported the absence of substantial publication bias for OS (**Figure 4A**), DFS (**Figure 4B**), and pCR (**Figure 4C**).

### 3.6 GRADE approach

Using the GRADE methodology, the quality of evidence was rated as low for OS, moderate for DFS, and very low for pCR. Detailed GRADE assessments are summarized in **Table 3**.

**Table 3:** GRADE rating of each outcome.

No. of study groups	Outcomes	HR/OR	95%CI	*I* ^2^; P value	Risk of bias	Inconsistency	Indirectness	Imprecision	Publication bias	Plausible confounding	Magnitude of effect	Dose-response gradient	GRADE
8	OS	1.64	1.27, 2.11	0%; P =0.92	No serious risk	No serious inconsistency	No serious indirectness	No serious imprecision	Undetected	Would not reduce effect	No	No	Low
4	DFS	2.29	1.54, 3.39	44%; P=0.08	No serious risk	No serious inconsistency	No serious indirectness	No serious imprecision	Undetected	Would not reduce effect	Yes	No	Moderate
2	PCR	1.51	1.24, 1.84	70%; P<0.0001	No serious risk	Serious inconsistency	No serious indirectness	No serious imprecision	Undetected	Would not reduce effect	No	No	Very low

## 4 Discussion

Inflammation plays a critical role in all stages of carcinogenesis, tumor progression, and resistance to anticancer therapies (40). Extensive evidence indicates that systemic inflammation is associated with poor survival in cancer patients, thereby supporting the clinical application of inflammatory markers as prognostic indicators (41). As a commonly used indicator of systemic inflammation, the PLR has been shown to be significantly associated with survival outcomes in colorectal, gastric, and hepatocellular cancers (42, 43). However, its prognostic value in BC remains controversial. Xue et al. reported that elevated PLR is associated with poorer OS, DFS, and pCR in BC patients (12), whereas Yuce et al. found no significant association between PLR and pCR (44).

Therefore, a meta-analysis involving 7,557 patients is conducted to evaluate the prognostic value of the PLR in breast cancer patients undergoing NACT. Our findings indicate that elevated PLR levels are significantly associated with shorter OS, reduced DFS, and lower pCR rates. In addition, sensitivity analyses further confirm the stability of these results. Egger’s test also reveals no evidence of publication bias. This finding is consistent with previous meta-analyses, which have shown that high PLR is significantly associated with lower pCR rates and poorer OS and DFS in breast cancer patients receiving NACT. Therefore, our study, based on a larger sample size, further validates these earlier findings and supports the potential role of PLR as an important biomarker for predicting treatment response to NACT in breast cancer patients.

In the subgroup analysis, PLR measured before NACT demonstrates greater prognostic value, whereas post-NACT PLR shows no significant association with breast cancer outcomes. Pre-NACT PLR reflects the intrinsic inflammatory state of the tumor microenvironment. Such chronic inflammation potentially influences tumor aggressiveness directly through mechanisms such as promoting angiogenesis and suppressing immune surveillance, thereby maintaining stable prognostic efficacy (45). Conversely, post-NACT PLR changes are influenced by chemotherapy and confounding factors, reflecting acute treatment-related stress responses, such as chemotherapy-induced neutropenia. These acute alterations have limited relevance to long-term prognosis, thus diminishing the predictive value of post-treatment PLR (46, 47). Therefore, clinical practice should prioritize pre-NACT PLR measurements for outcome prediction. In our meta-analysis, subgroup analyses by geographic region reveal no significant association between PLR and breast cancer prognosis in European or multicenter groups. The lack of statistical significance might result from the limited number of included studies. Additionally, genetic heterogeneity in populations outside Asia, such as differences between Northern and Southern European genetic backgrounds, may influence inflammatory responses. Furthermore, multicenter studies typically adhere to highly standardized treatment protocols, potentially masking the effects of PLR and generating false-negative results. Future international multicenter studies with standardized PLR measurement procedures are necessary to further validate the consistency of PLR’s prognostic value across diverse populations. Subgroup analysis based on PLR cutoff values indicates superior predictive efficacy in NACT-treated breast cancer patients when the cutoff value is ≥150 compared to <150. This finding suggests that future predictive models should ideally set PLR cutoff values at 150 or higher, or adjust them comprehensively based on specific patient characteristics such as tumor staging, treatment efficacy, therapeutic response, and age. Our findings indicate that cutoff values might contribute to heterogeneity observed in these analyses.

Although the exact biological pathways through which PLR influences breast cancer prognosis have yet to be fully elucidated, PLR has nonetheless shown promise as a predictive marker of responsiveness to NACT. PLR depends on platelet and lymphocyte levels, serving respectively as pro-tumor and anti-tumor indicators (21). Lymphocytes suppress breast cancer progression through immune surveillance mechanisms, including mediating cytotoxic apoptosis of tumor cells (48) and secreting anti-tumor factors such as interferon-γ (IFN-γ) and tumor necrosis factor-α (TNF-α) (49). High levels of TILs not only improve patient prognosis but also predict responses to NACT (50–53). Conversely, platelets accelerate cancer progression through a triple pro-tumor mechanism. First, platelets aggregate around tumor cells, forming a physical barrier against blood shear stress and immune attacks (54). Second, platelets secrete pro-angiogenic factors, such as vascular endothelial growth factor (VEGF), promoting tumor vascularization (55–57). Lastly, platelets trigger epithelial–mesenchymal transition (EMT), facilitating metastasis and hindering immune clearance (58). The disruption of lymphocyte–platelet balance elevates PLR, ultimately signaling poor breast cancer prognosis.

Although this meta-analysis offers a comprehensive summary of existing data, several limitations merit careful consideration. To begin with, most of the studies meeting the inclusion criteria for our analysis were conducted at single institutions and were predominantly based in Asian populations. Consequently, the findings should be interpreted cautiously within this specific geographic context. Generalization to patients in Europe, Africa, the Americas, or other regions may be inappropriate without further validation. Indeed, additional investigations are required to verify the prognostic value of PLR among breast cancer patients from non-Asian populations undergoing NACT. Secondly, the majority of the included investigations adopt a retrospective study design, as opposed to a prospective approach. Retrospective study designs are intrinsically susceptible to confounding variables, which may undermine both the accuracy and interpretability of the findings. Furthermore, inconsistency in PLR cutoff values across different studies poses another limitation. Some studies established cutoff values based on previous literature rather than employing ROC curve analyses. Even in studies utilizing ROC curve analysis, variability in blood sampling protocols, baseline hematological parameters, or timing of assessments may have resulted in inconsistent cutoff thresholds. Such variability could introduce selection bias into the meta-analysis. Therefore, future research would benefit from establishing standardized and universally accepted cutoff values for PLR to improve consistency and comparability across studies.

While our meta-analysis confirms PLR’s prognostic value in NACT-treated breast cancer, its biological interpretation warrants caution. The term inflammation oversimplifies a multifactorial process: elevated PLR may concurrently reflect platelet-mediated pro-tumorigenic pathways (e.g., VEGF-driven angiogenesis, EMT facilitation) and impaired lymphocyte-dependent immune surveillance (59–63). This mechanistic complexity underscores why PLR should not yet guide definitive clinical actions.

However, in the specific context of NACT, PLR offers practical utility. As pCR strongly correlates with survival (64, 65), a readily accessible biomarker predicting pCR failure (PLR ≥150) could help triage high-risk patients for advanced imaging or molecular profiling. This is particularly relevant in resource-constrained regions where genomic testing remains inaccessible. Future studies should integrate PLR with established biomarkers to build multimodal risk models rather than relying on isolated metrics.

## 5 Conclusion

Our meta-analysis demonstrates that elevated PLR significantly correlates with worse outcomes in breast cancer patients undergoing NACT, including reduced OS, shorter DFS, and lower pCR rates. These findings suggest PLR as a potentially valuable independent prognostic biomarker for informing clinical decisions regarding neoadjuvant treatment strategies. However, considering the limitations inherent in the included studies, further prospective research across diverse ethnic and geographical populations is necessary to validate these results.

## Data Availability

The original contributions presented in the study are included in the article/**Supplementary Material**. Further inquiries can be directed to the corresponding authors.

## References

[1] Bray F Laversanne M Sung H Ferlay J Siegel RL Soerjomataram I . Global cancer statistics 2022: GLOBOCAN estimates of incidence and mortality worldwide for 36 cancers in 185 countries. CA Cancer J Clin. (2024) 74:229–63. doi: 10.3322/caac.21834, PMID: 38572751

[2] Daly MB Pal T Maxwell KN Churpek J Kohlmann W AlHilli Z . NCCN guidelines^®^ Insights: genetic/familial high-risk assessment: breast, ovarian, and pancreatic, version 2.2024. J Natl Compr Canc Netw. (2023) 21:1000–10. doi: 10.6004/jnccn.2023.0051, PMID: 37856201

[3] Giaquinto AN Sung H Newman LA Freedman RA Smith RA Star J . Breast cancer statistics 2024. CA Cancer J Clin. (2024) 74:477–95. doi: 10.3322/caac.21863, PMID: 39352042

[4] Spring LM Fell G Arfe A Sharma C Greenup R Reynolds KL . Pathologic complete response after neoadjuvant chemotherapy and impact on breast cancer recurrence and survival: A comprehensive meta-analysis. Clin Cancer Res. (2020) 26:2838–48. doi: 10.1158/1078-0432.Ccr-19-3492, PMID: 32046998 PMC7299787

[5] Le-Petross HT McCall LM Hunt KK Mittendorf EA Ahrendt GM Wilke LG . Axillary ultrasound identifies residual nodal disease after chemotherapy: results from the American college of surgeons oncology group Z1071 trial (Alliance). AJR Am J Roentgenol. (2018) 210:669–76. doi: 10.2214/ajr.17.18295, PMID: 29381381 PMC5884109

[6] von Minckwitz G Untch M Blohmer JU Costa SD Eidtmann H Fasching PA . Definition and impact of pathologic complete response on prognosis after neoadjuvant chemotherapy in various intrinsic breast cancer subtypes. J Clin Oncol. (2012) 30:1796–804. doi: 10.1200/jco.2011.38.8595, PMID: 22508812

[7] Cortazar P Zhang L Untch M Mehta K Costantino JP Wolmark N . Pathological complete response and long-term clinical benefit in breast cancer: the CTNeoBC pooled analysis. Lancet. (2014) 384:164–72. doi: 10.1016/s0140-6736(13)62422-8, PMID: 24529560

[8] Xu W Chen X Deng F Zhang J Zhang W . and J tang predictors of neoadjuvant chemotherapy response in breast cancer: A review. Onco Targets Ther. (2020) 13:5887–99. doi: 10.2147/ott.S253056, PMID: 32606799 PMC7320215

[9] Şahin AB Cubukcu E Ocak B Deligonul A Oyucu Orhan S Tolunay S . Low pan-immune-inflammation-value predicts better chemotherapy response and survival in breast cancer patients treated with neoadjuvant chemotherapy. Sci Rep. (2021) 11:14662. doi: 10.1038/s41598-021-94184-7, PMID: 34282214 PMC8289916

[10] Zhang X Zhao W Yu Y Qi X Song L Zhang C . Clinicopathological and prognostic significance of platelet-lymphocyte ratio (PLR) in gastric cancer: an updated meta-analysis. World J Surg Oncol. (2020) 18:191. doi: 10.1186/s12957-020-01952-2, PMID: 32731872 PMC7391520

[11] Kubota K Shimizu A Notake T Masuo H Hosoda K Yasukawa K . Preoperative peripheral blood lymphocyte-to-monocyte ratio predicts long-term outcome for patients with pancreatic ductal adenocarcinoma. Ann Surg Oncol. (2022) 29:1437–48. doi: 10.1245/s10434-021-10848-8, PMID: 34664139

[12] Qi X Chen J Wei S Ni J Song L Jin C . Prognostic significance of platelet-to-lymphocyte ratio (PLR) in patients with breast cancer treated with neoadjuvant chemotherapy: a meta-analysis. BMJ Open. (2023) 13:e074874. doi: 10.1136/bmjopen-2023-074874, PMID: 37996220 PMC10668253

[13] Li F Wang Y Dou H Chen X Wang J . and M Xiao Association of immune inflammatory biomarkers with pathological complete response and clinical prognosis in young breast cancer patients undergoing neoadjuvant chemotherapy. Front Oncol. (2024) 14:1349021. doi: 10.3389/fonc.2024.1349021, PMID: 38380360 PMC10877026

[14] Fiste O Mavrothalassitis E Kokkalis A Anagnostakis M Gomatou G Kontogiannis A . Inflammation-related biomarkers as predictors of pathological complete response in early-stage breast cancer. Clin Transl Oncol. (2024) 27:2453–60. doi: 10.1007/s12094-024-03814-9, PMID: 39668275

[15] Zhu J Cheng J Ma Y Wang Y Zou Z Wang W . The value of inflammation-related indicators in chemotherapy efficacy and disease-free survival of triple-negative breast cancer. Eur J Med Res. (2025) 30:77. doi: 10.1186/s40001-025-02328-6, PMID: 39905567 PMC11792380

[16] Faur IF Dobrescu A Clim IA Pasca P Burta C Tarta C . Prognostic significance of peripheral blood parameters as predictor of neoadjuvant chemotherapy response in breast cancer. Int J Mol Sci. (2025) 26. doi: 10.3390/ijms26062541, PMID: 40141182 PMC11942583

[17] Wang H Huang Z Xu B Zhang J He P Gao F . The predictive value of systemic immune-inflammatory markers before and after treatment for pathological complete response in patients undergoing neoadjuvant therapy for breast cancer: a retrospective study of 1994 patients. Clin Transl Oncol. (2024) 26:1467–79. doi: 10.1007/s12094-023-03371-7, PMID: 38190034

[18] Chen X Cai Q Deng L Chen M Xu M Chen L . Association of inflammatory blood markers and pathological complete response in HER2-positive breast cancer: a retrospective single-center cohort study. Front Immunol. (2024) 15:1465862. doi: 10.3389/fimmu.2024.1465862, PMID: 39628488 PMC11611895

[19] Ma R Wei W Ye H Dang C Li K . and D Yuan A nomogram based on platelet-to-lymphocyte ratio for predicting pathological complete response of breast cancer after neoadjuvant chemotherapy. BMC Cancer. (2023) 23:245. doi: 10.1186/s12885-023-10703-x, PMID: 36918796 PMC10015959

[20] Kusama H Kittaka N Soma A Taniguchi A Kanaoka H Nakajima S . Predictive factors for response to neoadjuvant chemotherapy: inflammatory and immune markers in triple-negative breast cancer. Breast Cancer. (2023) 30:1085–93. doi: 10.1007/s12282-023-01504-y, PMID: 37782377

[21] Dan J Tan J Huang J Yuan Z Guo Y . Early changes of platelet−lymphocyte ratio correlate with neoadjuvant chemotherapy response and predict pathological complete response in breast cancer. Mol Clin Oncol. (2023) 19:90. doi: 10.3892/mco.2023.2686, PMID: 37854328 PMC10580258

[22] Page MJ McKenzie JE Bossuyt PM Boutron I Hoffmann TC Mulrow CD . The PRISMA 2020 statement: an updated guideline for reporting systematic reviews. BMJ. (2021) 372:n71. doi: 10.1136/bmj.n71, PMID: 33782057 PMC8005924

[23] Aanesen F Berg RC Jørgensen IL Mohr B Proper K . and L K Lunde Employment and mental health in the working age population: a protocol for a systematic review of longitudinal studies. Syst Rev. (2024) 13:197. doi: 10.1186/s13643-024-02613-1, PMID: 39054503 PMC11274751

[24] Higgins JP Thompson SG Deeks JJ . and D G Altman Measuring inconsistency in meta-analyses. BMJ. (2003) 327:557–60. doi: 10.1136/bmj.327.7414.557, PMID: 12958120 PMC192859

[25] Guyatt G Oxman AD Akl EA Kunz R Vist G Brozek J . GRADE guidelines: 1. Introduction-GRADE evidence profiles and summary of findings tables. J Clin Epidemiol. (2011) 64:383–94. doi: 10.1016/j.jclinepi.2010.04.026, PMID: 21195583

[26] Song D Li X Zhang X . Expression and prognostic value of ratios of platelet lymphocyte, neutrophil lymphocyte and lymphocyte monocyte in breast cancer patients. Am J Transl Res. (2022) 14:3233–9., PMID: 35702097 PMC9185042

[27] Corbeau I Thezenas S Maran-Gonzalez A Colombo PE Jacot W . and S guiu inflammatory blood markers as prognostic and predictive factors in early breast cancer patients receiving neoadjuvant chemotherapy. Cancers (Basel). (2020) 12:3–8. doi: 10.3390/cancers12092666, PMID: 32962003 PMC7564656

[28] Alan O Akin Telli T Aktas B Koca S Ökten IN Hasanov R . Is insulin resistance a predictor for complete response in breast cancer patients who underwent neoadjuvant treatment? World J Surg Oncol. (2020) 18:242. doi: 10.1186/s12957-020-02019-y, PMID: 32907593 PMC7488234

[29] Ma Y Zhang J . and X chen lymphocyte-to-monocyte ratio is associated with the poor prognosis of breast cancer patients receiving neoadjuvant chemotherapy. Cancer Manag Res. (2021) 13:1571–80. doi: 10.2147/cmar.S292048, PMID: 33623436 PMC7896736

[30] Asano Y Kashiwagi S Onoda N Noda S Kawajiri H Takashima T . Platelet-lymphocyte ratio as a useful predictor of the therapeutic effect of neoadjuvant chemotherapy in breast cancer. PLoS One. (2016) 11:e0153459. doi: 10.1371/journal.pone.0153459, PMID: 27472762 PMC4966926

[31] Acikgoz O Yildiz A Bilici A Olmez OF Basim P . and A Cakir Pretreatment platelet-to-lymphocyte ratio and neutrophil-to-lymphocyte ratio as a predictor of pathological complete response to neoadjuvant chemotherapy in patients with breast cancer: single center experience from Turkey. Anticancer Drugs. (2022) 33:1150–5. doi: 10.1097/cad.0000000000001389, PMID: 36206103

[32] Jiang C Zhang S Qiao K Xiu Y Yu X . and Y huang the pretreatment systemic inflammation response index as a useful prognostic factor is better than lymphocyte to monocyte ratio in breast cancer patients receiving neoadjuvant chemotherapy. Clin Breast Cancer. (2022) 22:424–38. doi: 10.1016/j.clbc.2022.03.003, PMID: 35428593

[33] Jin X Wang K Shao X . and J Huang Prognostic implications of the peripheral platelet-to-lymphocyte ratio and neutrophil-to-lymphocyte ratio in predicting pathologic complete response after neoadjuvant chemotherapy in breast cancer patients. Gland Surg. (2022) 11:1057–66. doi: 10.21037/gs-22-244, PMID: 35800742 PMC9253186

[34] Kim HY Kim TH Yoon HK . and A lee the role of neutrophil-lymphocyte ratio and platelet-lymphocyte ratio in predicting neoadjuvant chemotherapy response in breast cancer. J Breast Cancer. (2019) 22:425–38. doi: 10.4048/jbc.2019.22.e41, PMID: 31598342 PMC6769392

[35] Jiang C Lu Y Zhang S . and Y huang systemic immune-inflammation index is superior to neutrophil to lymphocyte ratio in prognostic assessment of breast cancer patients undergoing neoadjuvant chemotherapy. BioMed Res Int. (2020) 2020:7961568. doi: 10.1155/2020/7961568, PMID: 33381583 PMC7762645

[36] Graziano V Grassadonia A Iezzi L Vici P Pizzuti L Barba M . Combination of peripheral neutrophil-to-lymphocyte ratio and platelet-to-lymphocyte ratio is predictive of pathological complete response after neoadjuvant chemotherapy in breast cancer patients. Breast. (2019) 44:33–8. doi: 10.1016/j.breast.2018.12.014, PMID: 30611095

[37] Al Jarroudi O El Bairi K Abda N Zaimi A Jaouani L Chibani H . Neutrophil-to-lymphocyte and platelet-to-lymphocyte ratios as predictors of outcomes in inflammatory breast cancer. biomark Med. (2021) 15:1289–98. doi: 10.2217/bmm-2020-0717, PMID: 34486882

[38] Truffi M Sottotetti F Gafni N Albasini S Piccotti F Morasso C . Prognostic potential of immune inflammatory biomarkers in breast cancer patients treated with neoadjuvant chemotherapy. Cancers (Basel). (2022) 14. doi: 10.3390/cancers14215287, PMID: 36358706 PMC9658892

[39] Van Berckelaer C Vermeiren I Vercauteren L Rypens C Oner G Trinh XB . The evolution and prognostic role of tumour-infiltrating lymphocytes and peripheral blood-based biomarkers in inflammatory breast cancer patients treated with neoadjuvant chemotherapy. Cancers (Basel). (2021) 13. doi: 10.3390/cancers13184656, PMID: 34572883 PMC8471511

[40] Crusz SM Balkwill FR . Inflammation and cancer: advances and new agents. Nat Rev Clin Oncol. (2015) 12:584–96. doi: 10.1038/nrclinonc.2015.105, PMID: 26122183

[41] Zhou Q Dong J Sun Q Lu N Pan Y . and X Han Role of neutrophil-to-lymphocyte ratio as a prognostic biomarker in patients with breast cancer receiving neoadjuvant chemotherapy: a meta-analysis. BMJ Open. (2021) 11:e047957. doi: 10.1136/bmjopen-2020-047957, PMID: 34561257 PMC8475153

[42] Mei P Feng W Zhan Y . and X Guo Prognostic value of lymphocyte-to-monocyte ratio in gastric cancer patients treated with immune checkpoint inhibitors: a systematic review and meta-analysis. Front Immunol. (2023) 14:1321584. doi: 10.3389/fimmu.2023.1321584, PMID: 38090560 PMC10711042

[43] Yang XC Liu H Liu DC Tong C Liang XW . and R H Chen Prognostic value of pan-immune-inflammation value in colorectal cancer patients: A systematic review and meta-analysis. Front Oncol. (2022) 12:1036890. doi: 10.3389/fonc.2022.1036890, PMID: 36620576 PMC9813847

[44] Yuce E Karakullukcu S Bulbul H Alandag C Saygin I . and H Kavgaci The effect of the change in hemoglobin-albumin-lymphocyte-platelet scores occurring with neoadjuvant chemotherapy on clinical and pathological responses in breast cancer. Bratisl Lek Listy. (2023) 124:59–63. doi: 10.4149/bll_2023_009, PMID: 36519609

[45] Guthrie GJ Charles KA Roxburgh CS Horgan PG McMillan DC Clarke The systemic inflammation-based neutrophil-lymphocyte ratio SJ . experience in patients with cancer. Crit Rev Oncol Hematol. (2013) 88:218–30. doi: 10.1016/j.critrevonc.2013.03.010, PMID: 23602134

[46] Serhan CN . The resolution of inflammation: the devil in the flask and in the details. FASEB J. (2011) 25:1441–8. doi: 10.1096/fj.11-0502ufm, PMID: 21532053 PMC3228345

[47] Gajewski TF Schreiber H . and Y X Fu Innate and adaptive immune cells in the tumor microenvironment. Nat Immunol. (2013) 14:1014–22. doi: 10.1038/ni.2703, PMID: 24048123 PMC4118725

[48] Hiraoka K Miyamoto M Cho Y Suzuoki M Oshikiri T Nakakubo Y . Concurrent infiltration by CD8+ T cells and CD4+ T cells is a favourable prognostic factor in non-small-cell lung carcinoma. Br J Cancer. (2006) 94:275–80. doi: 10.1038/sj.bjc.6602934, PMID: 16421594 PMC2361103

[49] Mijic S . and C dabrosin platelet activation *in situ* in breasts at high risk of cancer: relationship with mammographic density and estradiol. J Clin Endocrinol Metab. (2021) 106:485–500. doi: 10.1210/clinem/dgaa820, PMID: 33180937

[50] Kotoula V Chatzopoulos K Lakis S Alexopoulou Z Timotheadou E Zagouri F . Tumors with high-density tumor infiltrating lymphocytes constitute a favorable entity in breast cancer: a pooled analysis of four prospective adjuvant trials. Oncotarget. (2016) 7:5074–87. doi: 10.18632/oncotarget.6231, PMID: 26506242 PMC4826267

[51] Ibrahim EM Al-Foheidi ME Al-Mansour MM . and G A Kazkaz The prognostic value of tumor-infiltrating lymphocytes in triple-negative breast cancer: a meta-analysis. Breast Cancer Res Treat. (2014) 148:467–76. doi: 10.1007/s10549-014-3185-2, PMID: 25361613

[52] Seo AN Lee HJ Kim EJ Kim HJ Jang MH Lee HE . Tumour-infiltrating CD8+ lymphocytes as an independent predictive factor for pathological complete response to primary systemic therapy in breast cancer. Br J Cancer. (2013) 109:2705–13. doi: 10.1038/bjc.2013.634, PMID: 24129232 PMC3833219

[53] Mao Y Qu Q Zhang Y Liu J Chen X . and K Shen The value of tumor infiltrating lymphocytes (TILs) for predicting response to neoadjuvant chemotherapy in breast cancer: a systematic review and meta-analysis. PLoS One. (2014) 9:e115103. doi: 10.1371/journal.pone.0115103, PMID: 25501357 PMC4264870

[54] Yang R Chang Q Meng X Gao N . and W Wang Prognostic value of Systemic immune-inflammation index in cancer: A meta-analysis. J Cancer. (2018) 9:3295–302. doi: 10.7150/jca.25691, PMID: 30271489 PMC6160683

[55] Egan K Crowley D Smyth P O’Toole S Spillane C Martin C . Platelet adhesion and degranulation induce pro-survival and pro-angiogenic signalling in ovarian cancer cells. PLoS One. (2011) 6:e26125. doi: 10.1371/journal.pone.0026125, PMID: 22022533 PMC3192146

[56] Kono SA Heasley LE Doebele RC . and D R Camidge Adding to the mix: fibroblast growth factor and platelet-derived growth factor receptor pathways as targets in non-small cell lung cancer. Curr Cancer Drug Targets. (2012) 12:107–23. doi: 10.2174/156800912799095144, PMID: 22165970 PMC3418220

[57] Klinger MH . and W Jelkmann Role of blood platelets in infection and inflammation. J Interferon Cytokine Res. (2002) 22:913–22. doi: 10.1089/10799900260286623, PMID: 12396713

[58] Floris G Richard F Hamy AS Jongen L Wildiers H Ardui J . Body mass index and tumor-infiltrating lymphocytes in triple-negative breast cancer. J Natl Cancer Inst. (2021) 113:146–53. doi: 10.1093/jnci/djaa090, PMID: 33152071 PMC7850533

[59] Salima S Sampeliling DG Permadi W Sasotya RMS Aziz MA Kurniadi A . Analysis of inflammation parameter value lymphocyte monocyte ratio (LMR), platelet lymphocyte ratio (PLR), and systemic inflammation response index (SIRI) to differentiate Malignant and benign ovarian tumors. BMC Res Notes. (2025) 18:328. doi: 10.1186/s13104-025-07330-z, PMID: 40721834 PMC12302886

[60] Maloney S Pavlakis N Itchins M Arena J Mittal A Hudson A . The prognostic and predictive role of the neutrophil-to-lymphocyte ratio (NLR), platelet-to-lymphocyte ratio (PLR), and lymphocyte-to-monocyte ratio (LMR) as biomarkers in resected pancreatic cancer. J Clin Med. (2023) 12. doi: 10.3390/jcm12051989, PMID: 36902776 PMC10004269

[61] Modica R Minotta R Liccardi A Cannavale G Benevento E . and A colao evaluation of neutrophil-to-lymphocyte ratio (NLR), platelet-to-lymphocyte ratio (PLR) and systemic immune-inflammation index (SII) as potential biomarkers in patients with sporadic medullary thyroid cancer (MTC). J Pers Med. (2023) 13. doi: 10.3390/jpm13060953, PMID: 37373942 PMC10304568

[62] Kim DH Jang SY . and B Keam Predictive value of early dynamic changes of NLR and PLR for the efficacy of immune checkpoint inhibitor in head and neck squamous cell carcinoma. Oral Surg Oral Med Oral Pathol Oral Radiol. (2024) 138:763–71. doi: 10.1016/j.oooo.2024.07.014, PMID: 39181857

[63] Knetki-Wróblewska M Grzywna A Krawczyk P Wojas-Krawczyk K Chmielewska I Jankowski T . Prognostic significance of neutrophil-to-lymphocyte ratio (NLR) and platelet-to-lymphocyte ratio (PLR) in second-line immunotherapy for patients with non-small cell lung cancer. Transl Lung Cancer Res. (2025) 14:749–60. doi: 10.21037/tlcr-24-675, PMID: 40248735 PMC12000957

[64] Moldoveanu D Hoskin TL Day CN Schulze AK Goetz MP . and J C Boughey Nodal pCR and overall survival following neoadjuvant chemotherapy for node positive ER+/Her2- breast cancer. Breast Cancer Res Treat. (2024) 203:419–28. doi: 10.1007/s10549-023-07152-2, PMID: 37878154 PMC11385785

[65] Pouwer AW Te Grootenhuis NC Hinten F de Bock GH van der Zee AGJ Melchers WJG . Prognostic value of HPV-PCR, p16 and p53 immunohistochemical status on local recurrence rate and survival in patients with vulvar squamous cell carcinoma. Virchows Arch. (2024) 484:985–94. doi: 10.1007/s00428-023-03690-8, PMID: 37938322 PMC11186908

