# Supplementary material for: Prognostic value of platelet to lymphocyte ratio (PLR) in breast cancer patients receiving neoadjuvant therapy: a systematic review and meta-analysis
Source: Front Immunol. 2025 Aug 20;16:1658571. doi: 10.3389/fimmu.2025.1658571 (PMC12405421; doi:10.3389/fimmu.2025.1658571)
Supplement: Supplementary file 1 [file Table1.docx]

| Supplementary Table S1. Detailed search strategy in four databases. | |
| --- | --- |
| Database | Search strategy |
| Pubmed | ((((("Blood Platelets"[Mesh]) OR (((((Blood Platelet) OR (Platelets)) OR (Platelet)) OR (Thrombocytes)) OR (Thrombocyte))) AND (("Lymphocytes"[Mesh]) OR (((Lymphocyte) OR (Lymphoid Cells)) OR (Lymphoid Cell)))) AND (Ratio)) AND (("Breast Neoplasms"[Mesh]) OR ((((((((((((((((((((Breast Neoplasm) OR (Breast Tumors)) OR (Breast Tumor)) OR (Breast Cancer)) OR (Cancer of Breast)) OR (Cancer of the Breast)) OR (Malignant Neoplasm of Breast)) OR (Breast Malignant Neoplasm)) OR (Breast Malignant Neoplasms)) OR (Malignant Tumor of Breast)) OR (Breast Malignant Tumor)) OR (Breast Malignant Tumors)) OR (Mammary Cancer)) OR (Mammary Cancers)) OR (Human Mammary Neoplasm)) OR (Human Mammary Neoplasms)) OR (Breast Carcinoma)) OR (Breast Carcinomas)) OR (Human Mammary Carcinomas)) OR (Human Mammary Carcinoma)))) AND (("Neoadjuvant Therapy"[Mesh]) OR ((((((((((((((((((((((((((((Neoadjuvant Therapies) OR (Neoadjuvant Treatment)) OR (Neoadjuvant Treatments)) OR (Neoadjuvant Chemotherapy)) OR (Neoadjuvant Chemotherapies)) OR (Neoadjuvant Chemotherapy Treatment)) OR (Neoadjuvant Chemotherapy Treatments)) OR (Neoadjuvant Chemoradiotherapy)) OR (Neoadjuvant Chemoradiotherapies)) OR (Neoadjuvant Chemoradiation)) OR (Neoadjuvant Chemoradiations)) OR (Neoadjuvant Chemoradiation Therapy)) OR (Neoadjuvant Chemoradiation Therapies)) OR (Neoadjuvant Chemoradiation Treatment)) OR (Neoadjuvant Chemoradiation Treatments)) OR (Neoadjuvant Systemic Therapy)) OR (Neoadjuvant Systemic Therapies)) OR (Neoadjuvant Systemic Treatment)) OR (Neoadjuvant Systemic Treatments)) OR (Neoadjuvant Radiotherapy)) OR (Neoadjuvant Radiotherapies)) OR (Neoadjuvant Radiation)) OR (Neoadjuvant Radiations)) OR (Neoadjuvant Radiation Therapy)) OR (Neoadjuvant Radiation Therapies)) OR (Neoadjuvant Radiation Treatment)) OR (Neoadjuvant Radiation Treatments)) OR (Neoadjuvant))) |
| Embase | ((Blood Platelets or (Blood Platelet or Platelets or Platelet or Thrombocytes or Thrombocyte)) and (Lymphocytes or (Lymphocyte or Lymphoid Cells or Lymphoid Cell)) and Ratio and (Breast Neoplasms or (Breast Neoplasm or Breast Tumors or Breast Tumor or Breast Cancer or Cancer of Breast or Cancer of the Breast or Malignant Neoplasm of Breast or Breast Malignant Neoplasm or Breast Malignant Neoplasms or Malignant Tumor of Breast or Breast Malignant Tumor or Breast Malignant Tumors or Mammary Cancer or Mammary Cancers or Human Mammary Neoplasm or Human Mammary Neoplasms or Breast Carcinoma or Breast Carcinomas or Human Mammary Carcinomas or Human Mammary Carcinoma)) and (Neoadjuvant Therapy or (Neoadjuvant Therapies or Neoadjuvant Treatment or Neoadjuvant Treatments or Neoadjuvant Chemotherapy or Neoadjuvant Chemotherapies or Neoadjuvant Chemotherapy Treatment or Neoadjuvant Chemotherapy Treatments or Neoadjuvant Chemoradiotherapy or Neoadjuvant Chemoradiotherapies or Neoadjuvant Chemoradiation or Neoadjuvant Chemoradiations or Neoadjuvant Chemoradiation Therapy or Neoadjuvant Chemoradiation Therapies or Neoadjuvant Chemoradiation Treatment or Neoadjuvant Chemoradiation Treatments or Neoadjuvant Systemic Therapy or Neoadjuvant Systemic Therapies or Neoadjuvant Systemic Treatment or Neoadjuvant Systemic Treatments or Neoadjuvant Radiotherapy or Neoadjuvant Radiotherapies or Neoadjuvant Radiation or Neoadjuvant Radiations or Neoadjuvant Radiation Therapy or Neoadjuvant Radiation Therapies or Neoadjuvant Radiation Treatment or Neoadjuvant Radiation Treatments or Neoadjuvant))).af. |
| Web of Science | (((((Blood Platelets) OR (((((Blood Platelet) OR (Platelets)) OR (Platelet)) OR (Thrombocytes)) OR (Thrombocyte))) AND ((Lymphocytes) OR (((Lymphocyte) OR (Lymphoid Cells)) OR (Lymphoid Cell)))) AND (Ratio)) AND ((Breast Neoplasms) OR ((((((((((((((((((((Breast Neoplasm) OR (Breast Tumors)) OR (Breast Tumor)) OR (Breast Cancer)) OR (Cancer of Breast)) OR (Cancer of the Breast)) OR (Malignant Neoplasm of Breast)) OR (Breast Malignant Neoplasm)) OR (Breast Malignant Neoplasms)) OR (Malignant Tumor of Breast)) OR (Breast Malignant Tumor)) OR (Breast Malignant Tumors)) OR (Mammary Cancer)) OR (Mammary Cancers)) OR (Human Mammary Neoplasm)) OR (Human Mammary Neoplasms)) OR (Breast Carcinoma)) OR (Breast Carcinomas)) OR (Human Mammary Carcinomas)) OR (Human Mammary Carcinoma)))) AND ((Neoadjuvant Therapy) OR ((((((((((((((((((((((((((((Neoadjuvant Therapies) OR (Neoadjuvant Treatment)) OR (Neoadjuvant Treatments)) OR (Neoadjuvant Chemotherapy)) OR (Neoadjuvant Chemotherapies)) OR (Neoadjuvant Chemotherapy Treatment)) OR (Neoadjuvant Chemotherapy Treatments)) OR (Neoadjuvant Chemoradiotherapy)) OR (Neoadjuvant Chemoradiotherapies)) OR (Neoadjuvant Chemoradiation)) OR (Neoadjuvant Chemoradiations)) OR (Neoadjuvant Chemoradiation Therapy)) OR (Neoadjuvant Chemoradiation Therapies)) OR (Neoadjuvant Chemoradiation Treatment)) OR (Neoadjuvant Chemoradiation Treatments)) OR (Neoadjuvant Systemic Therapy)) OR (Neoadjuvant Systemic Therapies)) OR (Neoadjuvant Systemic Treatment)) OR (Neoadjuvant Systemic Treatments)) OR (Neoadjuvant Radiotherapy)) OR (Neoadjuvant Radiotherapies)) OR (Neoadjuvant Radiation)) OR (Neoadjuvant Radiations)) OR (Neoadjuvant Radiation Therapy)) OR (Neoadjuvant Radiation Therapies)) OR (Neoadjuvant Radiation Treatment)) OR (Neoadjuvant Radiation Treatments)) OR (Neoadjuvant))) (Topic) |
| Chochrane | ((Blood Platelets or (Blood Platelet or Platelets or Platelet or Thrombocytes or Thrombocyte)) and (Lymphocytes or (Lymphocyte or Lymphoid Cells or Lymphoid Cell)) and Ratio and (Breast Neoplasms or (Breast Neoplasm or Breast Tumors or Breast Tumor or Breast Cancer or Cancer of Breast or Cancer of the Breast or Malignant Neoplasm of Breast or Breast Malignant Neoplasm or Breast Malignant Neoplasms or Malignant Tumor of Breast or Breast Malignant Tumor or Breast Malignant Tumors or Mammary Cancer or Mammary Cancers or Human Mammary Neoplasm or Human Mammary Neoplasms or Breast Carcinoma or Breast Carcinomas or Human Mammary Carcinomas or Human Mammary Carcinoma)) and (Neoadjuvant Therapy or (Neoadjuvant Therapies or Neoadjuvant Treatment or Neoadjuvant Treatments or Neoadjuvant Chemotherapy or Neoadjuvant Chemotherapies or Neoadjuvant Chemotherapy Treatment or Neoadjuvant Chemotherapy Treatments or Neoadjuvant Chemoradiotherapy or Neoadjuvant Chemoradiotherapies or Neoadjuvant Chemoradiation or Neoadjuvant Chemoradiations or Neoadjuvant Chemoradiation Therapy or Neoadjuvant Chemoradiation Therapies or Neoadjuvant Chemoradiation Treatment or Neoadjuvant Chemoradiation Treatments or Neoadjuvant Systemic Therapy or Neoadjuvant Systemic Therapies or Neoadjuvant Systemic Treatment or Neoadjuvant Systemic Treatments or Neoadjuvant Radiotherapy or Neoadjuvant Radiotherapies or Neoadjuvant Radiation or Neoadjuvant Radiations or Neoadjuvant Radiation Therapy or Neoadjuvant Radiation Therapies or Neoadjuvant Radiation Treatment or Neoadjuvant Radiation Treatments or Neoadjuvant))).af. |
