# Supplementary material for: Prognostic value of platelet to lymphocyte ratio (PLR) in breast cancer patients receiving neoadjuvant therapy: a systematic review and meta-analysis
Source: Front Immunol. 2025 Aug 20;16:1658571. doi: 10.3389/fimmu.2025.1658571 (PMC12405421; doi:10.3389/fimmu.2025.1658571)
Supplement: Supplementary file 2 [file Table2.docx]

| Supplementary Table S2. Quality evaluation of the eligible studies with Newcastle–Ottawa scale. | | | | | | | | | |
| --- | --- | --- | --- | --- | --- | --- | --- | --- | --- |
| Study | Selection | | | | Comparability | | Outcome | | |
|  | Representative-ness | Selection of  non-exposed | Ascertainment  of exposure | Outcome not present at start | Comparability on most important factors | Comparability on other risk factors | Assessment of outcome | Long enough follow-up (median≥1 year) | Adequacy  (completeness) of follow-up |
| Li 2024 | - | * | * | * | - | - | * | * | * |
| Chen 2024 | * | * | * | * | - | - | * | * | * |
| Graziano 2019 | * | * | * | * | - | * | * | * | * |
| Dan 2023 | * | * | * | * | - | - | * | * | * |
| Van Berckelaer 2021 | * | - | * | * | - | - | * | * | * |
| Song 2022 | * | * | * | * | - | * | * | * | * |
| Fiste 2024 | * | - | * | * | - | - | * | * | * |
| Corbeau 2020 | * | * | * | * | - | - | * | * | * |
| Alan 2020 | * | * | * | * | - | - | * | * | * |
| Şahin 2021 | * | * | * | * | - | - | * | * | * |
| Ma 2021 | * | * | * | * | - | - | * | * | * |
| Al Jarroudi 2021 | * | * | * | * | - | - | * | * | * |
| Ma 2023 | * | * | * | * | - | - | * | * | * |
| Asano 2016 | * | * | * | * | - | - | * | * | * |
| Kusama 2023 | * | * | * | * | - | - | * | * | * |
| Wang 2024 | * | * | * | * | - | - | * | * | * |
| Acikgoz 2022 | * | * | * | * | - | - | * | * | * |
| Jiang 2022 | * | * | * | * | - | * | * | * | * |
| Jin 2022 | * | * | * | * | - | * | * | * | * |
| Truffi 2022 | * | * | * | * | - | - | * | * | * |
| Faur 2025 | * | * | * | * | - | - | * | * | * |
| Kim 2019 | * | * | * | * | - | * | * | * | * |
| Jiang 2020 | * | * | * | * | * | * | * | * | * |
| Zhu 2025 | * | * | * | * | - | - | * | * | * |
| *indicates criterion met; - indicates significant of criterion not met. | | | | | | | | | |
